# Progress in Surgery

**Published:** 1902-02-22

**Authors:** 


### Progress in Surgery.

### OTOLOGY.

**The Production of Anæsthesia in the Ear.**—Homer Dupuy (New Orleans) [1] has made extensive trial of Dr. Albert Gray's solution of cocaine in equal parts of aniline oil and absolute alcohol in aural practice. He considers it almost impossible to incise the drum membrane at the best point for drainage with the aid of ordinary cocaine solutions, for they do not penetrate the membrane sufficiently to secure anæsthesia, and therefore all the patients move at the important moment. In his first three of 30 cases Dupuy used the above solution in the strength of 10 per cent., and with it obtained complete anæsthesia for incision of the drum. In his fourth and fifth cases he was not so successful; therefore, in his next 10 cases, he used a solution of the strength of 15 per cent. In a series of 14 cases he used a 20 per cent. solution. Altogether 50 cases were observed, and in all, with the exceptions mentioned above, and one other, the anæsthesia was complete. Similarly the solution was found efficient in two cases in which the drum was not inflamed. Only once were any untoward symptoms noticed, and they were not serious. The solution dehydrates the tissues and then penetrates the interstices until the nerve terminations in the deeper layers are reached. The writer recommends, as a preliminary step before using the cocaine solution, that the loose epithelial tissue on the drum membrane be removed with the aid of a hydrogen dioxide solution, followed by ordinary syringing. Also the meatus, it is said, should be filled with the solution, and the fluid must be allowed to remain in it 10 or 15 minutes. Before incising the drum may be touched to see if it is insensitive. The 20 per cent. solution is considered the most satisfactory. St. Clair Thomson [2] has recorded a case where the use of the above solution, of 10 per cent. strength, gave rise to toxic symptoms. The patient, an adult, had furunculosis of the meatus, and applied the solution on cotton wool at bedtime. At 5 A.M. it was again used. At 7.30 A.M., while still in bed, blueness of the fingers was accidentally noticed. The face was also blue and the pulse weak. Examination of the heart showed enlargement of the left ventricle. These effects were attributed to the toxic effect of the aniline oil on the red corpuscles. In the course of the day the disturbances gradually passed off. The writer refers to a report on the cases of ten children who were seized with prostration, pallor, and blueness soon after wearing yellow socks dyed with a pigment containing 90 per cent. of aniline. Also to the fact that workers in aniline works have been known to suffer similarly. The symptoms are not unlike those of antipyrin poisoning.

**Deafness from Rarefaction of the Labyrinthine Capsule.**—Professor Siebenmann [3] has given a minute report of a case of the above kind. He contends that excessive rarefaction of the bony capsule is sufficient, without involvement of the bony nerve-canals, to produce a marked diminution of bone conduction. In the case described deafness gradually developed severely in the right ear, slightly in the left. The Rinne test was positive and the Weber negative. Post mortem on both sides there were found areas of rarefaction in the bony capsule of the semicircular canals, vestibule, and cochlea. On the inner and external surfaces of the margin of the oval window there were osteophytes, and there was some ossification in the cartilaginous covering of the stapes. The diminished bone conduction is attributed to variation in density of the labyrinthine fluid. The internal administration of phosphorus is suggested as a mode of treatment.

**The Effects of Tinnitus Aurium upon Articulation and Vocalisation.**—Carl Seiler, New York,[4] has recorded some results of his observations upon the effects of cinchonism, with its aural symptom of tinnitus, upon articulation and vocalisation, and also the allied effects of non-subjective external noises. It was very difficult to get definite data in regard to the loudness and pitch both of the subjective aural sensations and of the external noises which interfered with articulation. The author used a complete set of Rudolph Kœnig's test tuning-forks and rods. He found that the ordinary tinnitus of middle-ear disease never transgressed the limits of pitch between "d-1," 297 vibrations as the lowest point, to the "f-2," 704 vibrations as the highest, and that these noises had no appreciable effect upon articulation, but that they materially affected the perception of sounds which had the same or nearly the same number of vibrations per second. The noises of so called cinchonism, as produced by drugs such as quinine, salicylate of sodium, alcohol, ether, and others, Seiler found to vary from as low as "g-3" (1,584 vibrations) to as high as I-4 (3,960 vibrations). The noises were composite, sometimes blended, forming a chord with a fundamental tone constant in pitch, but varying in pitch with the drug used, being usually lowest with quinine, and highest with salicylate of sodium. Another of the author's observations was that any external composite noise of high pitch, such as that of hissing steam or revolving grindstones, interfered with the pronunciation of those consonants possessing a high-pitched sound, such as "th," "s," "sh," and "z," and also made those consonants very difficult to recognise by the ear. This fact, which is familiar to steam engineers, grinders, and others, is easily explained by the laws of interference of sound waves of the same or nearly the same length with one another. The noises of cinchonism materially interfere with the pronunciation of the consonants mentioned, and even alters the quality of the high-pitched vowel sounds.

**Mastoiditis.**—F. Waterhouse [5] points out that in very acute inflammatory processes with a high temperature and pulse-rate, severe pain and constitutional disturbance, and swelling over the mastoid, everyone would advise that the mastoid should be opened. The cases where a difference of opinion may exist are those in which there is only a moderate discharge of pus, and the symptoms are not very obtrusive. He recommends operation when chronic otorrhœa is associated with (1) continuous or frequent pain in ear, mastoid, or side of head, particularly if they are aggravated by pressure on the mastoid; (2) total loss of hearing, with frequent attacks of giddiness, suggesting involvement of the internal ear; (3) caries or necrosis; (4) polypi and granulations which recur and resist careful treatment with biniodide and spirit drops; (5) caseous deposits and cholesteatomata; (6) facial paralysis; (7) distinct symptoms of intracranial abscess, meningitis, or sinus thrombosis. The writer states that manifestly thorough exploration of the mastoid must be carried out before, or at the same time as, the operation for any intracranial complication of purulent otorrhœa. To leave the paths by which the organisms enter uneradicated will render recovery doubtful, and expose the patient to fresh attacks (MacEwen). E. B. Dench [6] advises that an exploratory incision be made on the mastoid in doubtful cases. He believes that spontaneous recovery is impossible if the mastoid inflammation has produced a fluctuant swelling behind the auricle. In a doubtful case delay may lead to the most serious complications. Dench believes that germs may lie dormant for an indefinite period in the mastoid process, and then become virulent again at any time from some exciting cause. He therefore believes that in every case in which there is any evidence of mastoid involvement it is safer for the patient to have an immediate operation rather than to have attempts made to abort the inflammatory process by the local abstraction of blood, the application of cold, and kindred measures. William H. Dudley [7] states that there is a variety of acute mastoiditis, usually following chronic purulent otitis, in which the symptoms and signs, the swelling, tinnitus, and pain, are only moderate in degree, yet, given time, the disease may extend and threaten the patient's life through the development of complications. He considers the dangers attending mastoiditis not operated upon in the early stages have not been sufficiently impressed on the profession. B. A. Randall, commenting on the above communication, said he believed that many of the mastoid inflammations were not purulent, and did not need operative intervention; at least four out of five cases of redness, swelling, and tenderness of the mastoid, in his experience, did not require operation, but would clear up under proper medication and rest.

[1] Laryngoscope, July 1901, p. 60. [2] Abst. Laryngoscope, Aug. 1901, p. 169. [3] Abst. Jour. L. R. & O., July 1901, p. 377. [4] Med. Record, Oct. 26, 1901, p. 656. [5] Edin. Med. Jour., Sept. 1901, p. 223. [6] Abst. Med. Record, Oct. 26, 1901, p. 668. [7] Med. Record, Oct. 12, 1901, p. 590.

